# On the Low Reliability of Sunk Cost Vignettes

**DOI:** 10.3390/brainsci15080808

**Published:** 2025-07-28

**Authors:** Michał Białek, Emilia Biesiada

**Affiliations:** Institute of Psychology, University of Wroclaw, 50-527 Wrocław, Poland; emilia.biesiada@uwr.edu.pl

**Keywords:** sunk cost bias, measurement reliability, construct validity, decision-making, neuroimaging

## Abstract

**Background/Objectives:** Sunk cost bias—continuing failing endeavours due to prior investments—is among the most studied decision-making biases. Despite decades of vignette-based research, these measures lack systematic psychometric validation. We examined whether widely-used sunk cost scenarios reliably measure the same psychological construct. **Methods:** Across two experiments (N = 395), we tested established sunk cost vignettes, including classic scenarios from Arkes and Blumer (1985). English-speaking participants from Prolific Academic completed vignettes alongside cognitive reflection and social desirability measures. We assessed internal consistency and intercorrelations between scenarios. **Results:** Internal consistency was consistently poor (ω = 0.14–0.57) with weak intercorrelations between scenarios. Even highly similar vignettes correlated only moderately. External validity was problematic, showing inconsistent relationships with cognitive reflection and social desirability across vignettes. **Conclusions:** These measurement failures have critical implications for neuroimaging research, where unreliable behavioural measures may be mistaken for genuine neural differences. The field needs systematic categorization of scenarios to identify which vignettes engage specific psychological processes and neural circuits, enabling more targeted theoretical development.

## 1. Introduction

Sunk cost bias—the tendency to continue investing in failing endeavours based on prior investments—represents one of the most studied biases in decision-making research [1,2,3]. From an economic perspective, rational decision-making should ignore sunk costs and focus solely on future costs and benefits. However, people systematically violate this principle across diverse contexts—from personal decisions about concert attendance to corporate investments in failing projects. Understanding this phenomenon is crucial for both theoretical advancement and practical applications, as it underlies costly errors in business investments [4], policy decisions [5], and health choices [6]. Recent research continues to demonstrate its widespread impact across domains, from corporate pricing strategies and consumer behaviour [7] to scientific research project management [8].

Sunk cost vignettes serve as fundamental tools for understanding the psychological and neural mechanisms underlying these decisions. While individual vignettes may predict some real-world behaviours, their primary scientific value lies in enabling researchers to develop unified theoretical accounts of when, why, and how sunk cost effects emerge. This theoretical goal requires that vignettes measure the same underlying psychological construct—an assumption that has rarely been tested empirically.

If different scenarios systematically cue different psychological motives to honour sunk costs—such as loss aversion (reluctance to accept losses), commitment consistency (maintaining prior decisions for self-concept reasons), or social signalling (appearing persistent to others)—then treating them as interchangeable measures will fundamentally mislead scientific understanding. Researchers using different vignettes would unknowingly study distinct psychological phenomena while believing they investigate the same construct, preventing the development of coherent theoretical frameworks. Researchers in the field are indeed aware that sunk cost effects might stem from different psychological sources. However, the field has continued using vignettes interchangeably, perhaps hoping that any underlying differences would average out or prove inconsequential.

Neuroimaging studies identified key brain regions involved in sunk cost decisions, with consistent evidence pointing to the ventromedial prefrontal cortex (vmPFC) supporting goal commitment and dorsolateral prefrontal cortex (dlPFC) implementing resource conservation norms [9,10]. Cross-species research has further revealed that mice, rats, and humans demonstrate similar sunk cost sensitivity in foraging tasks, suggesting evolutionarily conserved neural mechanisms [11].

However, these neuroscientific advances depend critically on valid behavioural measurement. Progress in sunk cost research may be constrained by a largely unrecognised measurement problem: the vignettes used to assess this bias appear to measure different constructs despite ostensibly tapping the same psychological phenomenon. If vignettes with poor convergent validity recruit different psychological processes, then attempts to build unified theories of sunk cost bias will fail because researchers are actually studying multiple distinct phenomena. For neuroimaging investigations, inconsistent behavioural measures will produce divergent neural activation patterns across studies—not because of genuine theoretical disagreements, but because different vignettes activate different psychological and neural mechanisms.

For example, insula activity strongly mediated personality-based sunk cost relationships [12], while others suggested vmPFC–dlPFC interactions as central to the effect [9]. Rather than reflecting competing theoretical accounts, these different neural mechanisms may simply reflect the use of vignettes that tap distinct psychological processes. Without reliable measurement that demonstrates construct validity, the field cannot distinguish between genuine theoretical differences and measurement artefacts.

Despite decades of sunk cost research aimed at understanding the psychological mechanisms underlying these decisions, the psychometric properties of common measurement approaches have received surprisingly little systematic attention. This oversight is particularly problematic because vignette-based measures serve as the primary tools for theory development—they enable researchers to test competing psychological accounts, identify neural mechanisms, and develop interventions. If these tools lack reliability and construct validity, scientific progress becomes impossible regardless of how well individual vignettes might predict specific real-world behaviours.

These measurement challenges extend beyond individual studies to fundamental questions about construct validity. If sunk cost measurement tools conflate multiple decision processes with distinct neural substrates, researchers will struggle to identify unified explanations for the phenomenon or develop effective interventions. Arkes and Blumer [1] explicitly aimed to demonstrate a general decision-making bias that would manifest consistently across diverse contexts, presenting their vignettes as equivalent measures of the same underlying irrationality. However, this fundamental assumption has rarely been tested empirically. Even though researchers typically agree on using established vignettes, we demonstrate that this consensus may be problematic, as scenarios fail to consistently measure the same preference within individuals.

### Overview of the Studies

To maintain compatibility with most papers in the sunk cost literature, in this project we adopt a behavioural definition: sunk cost effects occur when people continue investing in failing endeavours because of prior investments that cannot be recovered, despite superior alternative options being available. This definition captures the core phenomenon that most researchers study while remaining agnostic about underlying psychological mechanisms.

All vignettes we tested seemingly fit this definition—they present scenarios where rational economic analysis suggests abandoning prior investments in favour of better alternatives. The ski trip vignette offers a better vacation option, the airline scenarios present superior competitors, and the TV dinner problem provides identical meals at different prior costs. Each scenario should theoretically elicit the same irrational tendency to honour sunk costs if this represents a unified psychological bias.

Across two experiments (N = 395), we examined the reliability and validity of established sunk cost scenarios. We found consistently poor internal consistency (ω = 0.14–0.57) and weak intercorrelations between scenarios, which revealed that these apparently equivalent scenarios tap different psychological processes. The ski trip may primarily engage loss aversion (reluctance to waste money), the airline scenarios might activate professional decision-making norms, and social versions of the vignettes could trigger reputation management concerns. While all produce behaviour that fits our working definition, the underlying psychology varies systematically.

External validity was equally problematic, with inconsistent relationships to cognitive reflection across experiments. These findings reveal fundamental measurement problems that may have impeded theoretical progress and compromised neuroimaging interpretations for decades.

Our findings provide empirical confirmation that the field’s intuitions about multiple underlying mechanisms are correct and have important consequences for measurement validity. The poor intercorrelations between established vignettes suggest that what researchers have long assumed to be alternative manifestations of the same psychological construct may actually represent distinct phenomena. This affects not just behavioural prediction, but also the fundamental scientific goal of understanding the psychology and neuroscience of persistence in failing endeavours. Without reliable measures that demonstrate construct validity, researchers cannot build cumulative knowledge about the mechanisms underlying sunk cost decisions.

## 2. Experiment 1

The current manuscript is a by-product of a project we started several years ago. We initially planned to investigate adaptive functions of sunk cost behaviour, such as reputation building [13,14,15] and moral signalling [16]. However, such research requires reliable individual difference measures. We therefore began by examining the psychometric properties of established sunk cost scenarios from Arkes and Blumer [1], planning to correlate individual differences with theoretically relevant measures if adequate reliability was achieved.

We planned to correlate the propensity to sunk cost with two measures: one confirmatory, aiming at replicating the well-known findings of honouring sunk-cost being negatively correlated to cognitive reflection [17,18] and an exploratory measure of social desirability—expecting a positive correlation between honouring sunk cost and people’s inclination to present themselves in a favourable light by endorsing socially acceptable behaviours and denying socially unacceptable ones. To meet these goals, we used the cognitive reflection test [19] and the Marlowe–Crowne social desirability scale [20].

All data and materials for this study and the second study are posted in a public repository https://osf.io/ubzv2/?view_only=63adfcdc96924ad68d814e086e816053 (accessed on 27 July 2025)

### 2.1. Participants

We analysed the data of 198 English native speakers, recruited from Prolific Academic using the Qualtrics survey platform. Informed consent was obtained from all subjects involved in the study. The sample consisted of 65 males, 124 females, and 9 participants who self-classified as “other”. Their average age was 40.6 (SD = 14.5, range 19–79).

We rejected data from an additional 3 participants, who reported having previously died of a heart attack while watching Netflix, suggesting their lack of attentiveness to the questions asked.

### 2.2. Materials

Participants responded to all 7 vignettes describing sunk cost decisions using a yes/no scale. The vignettes were presented in random order (order was not recorded for analysis). Decisions were hypothetical, with no monetary or other incentives provided. Some of the items were highly similar versions of a problem, asking a participant to either decide or recommend a solution. We selected these widely used scenarios from the seminal Arkes and Blumer [1] paper, expecting them to be representative of the subsequently emerging research tradition. An example item is as follows:

*The Acme Airlines Company has received a suggestion from one of its employees. The suggestion is to use the last 1 million dollars of research funds to build a plane that would not be detected by conventional radar, in other words, a radar-blank plane. However, another firm has just begun marketing a plane that cannot be detected by radar. Also, it is apparent that their plane is much faster and far more economical than the plane your company could build. The question is, should you invest the last million dollars of your research funds to build the radar-blank plane proposed by your employee?*

Next, participants responded to a 13-item version of the Marlowe–Crowne social desirability scale [20], using yes/no response options. Then they responded to the 3-item long Cognitive Reflection Test (CRT) [19]. The test presents participants with mathematical tasks, deliberately designed to lure them into intuitively appealing incorrect responses. An example item is the famous bat-and-ball problem:

*A bat and a ball cost $1.10 in total. The bat costs $1.00 more than the ball. How much does the ball cost?*

Finally, participants reported their knowledge about the economy (with 5 choice options, ranging from “I have studied/am studying subject related to economy in my university” to “My knowledge about economy is limited and therefore I don’t feel the need to expand it”); their “knowledge about any of the subjects mentioned in the study (cost of ski trips, working in an airline/printing company)” using a 5-point scale; and their financial situation (from 1—“Very satisfying (I have financial security, can afford making savings and don’t need to deny myself some pleasures and/or pursuing my dreams)” to 5—“Very unsatisfying (I don’t have any savings and can’t afford making them; my sole focus is on staying afloat, so I almost always have to deny myself pleasures and sometimes have to choose between satisfying one need or another, e.g., buying food vs. buying sanitary products)”.

### 2.3. Results

We started our investigation by exploring the reliability of a scale constructed of the seven items measuring the sunk cost bias. The scale included four versions of a vignette about a stealth plane (“Airline company” and “Acme”), one in each pair asking for an individual decision and the other for a recommendation to an actual decision-maker.

However, we observed that its reliability was unsatisfactory (ω = 0.57). Table 1, which presents the item means and a reliability analysis, shows that there was not a single item that could be removed which would significantly improve the scale’s reliability. Surprisingly, among the best-to-remove candidates were the two famous vignettes developed by the Nobel Prize laureate Richard Thaler—the ski trip to Michigan and the TV dinner problem.

**Figure 1:**
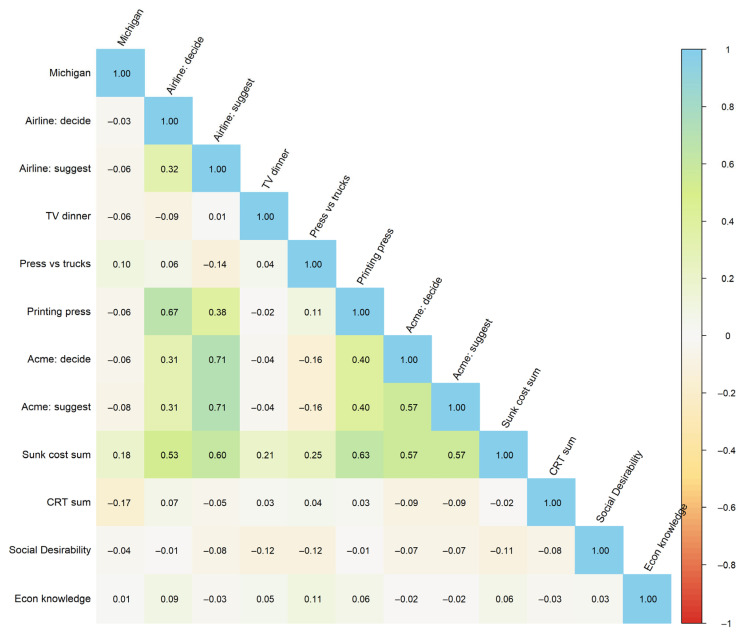
Intercorrelations heatmap for Experiment 1. Note: Correlations are measured by Kendall’s Tau-b.

Given these reliability problems, we examined each scenario’s correlations to other items and to external factors (Figure 1). The idea was that the items, if testing a true underlying construct, should show a similar pattern of correlations with the CRT and Marlowe–Crowne methods. We calculated intercorrelations using Kendall’s tau-b rather than Pearson correlations because our binary response format violated assumptions of parametric correlations, and Kendall’s tau is more robust to tied values and non-normal distributions common in vignette data.

Also, this analysis made the measurements look grim. Despite high similarities in their content, the scenarios correlated only weakly with each other and inconsistently with external measures. For example, there was only one significant correlation between the CRT results and sunk cost scenarios: the Michigan ski trip problem, which was uncorrelated with the sum score of the sunk costs scale. None of the scenarios correlated with the Social Desirability scale.

### 2.4. Discussion

We observed an unexpected pattern of low internal correlations between the items designed to measure the propensity to honour sunk costs. Some of the vignettes were very similar in their context (building a stealth plane) and all are popular in the literature. Particularly surprising was the poor performance of highly cited scenarios such as the Michigan ski trip and TV dinner vignettes, which showed among the lowest item–rest correlations despite being foundational to the sunk cost literature. This pattern suggests that even the most established measures in the field may not reliably assess the same psychological construct.

One could argue that the low reliability of a scale constructed of those items resulted from the carelessness of our participants. Yet, there are several reasons to reject this as an explanation: they spent a reasonable amount of time reading and deciding on each scenario (about 30–40 s), they performed quite well in the CRT, and almost all passed the attention check we performed to filter out inattentive participants in the past [21,22]. For exploratory reasons, we re-ran the reliability analysis, excluding participants who completed the entire study in less than 200 s (N = 196, ω = 0.57), 250 s (N = 183, ω = 0.56), or 300 s (N = 175, ω = 0.57). The reliability remained similarly low.

The literature on sunk cost bias created many more vignettes that study the same phenomenon, which are not as similar to each other as the ones we selected for this project. In a subsequent study, we used a greater set of scenarios, searching for a subset of these that would create a coherent scale.

## 3. Experiment 2

Having established reliability problems with the most canonical sunk cost vignettes, we next investigated whether a broader, more diverse set of scenarios might reveal better psychometric properties. Continuing with the initial goal of the project (i.e., testing the social context of honouring sunk costs), we selected a set of 12 scenarios, 6 that were already used in the literature, and 6 that we constructed based on the template (Table 2). We hoped that the new set of vignettes would show better intercorrelations than in Experiment 1 due to the inclusion of more diverse contexts, and that social framing might increase correlations by activating consistent reputation management concerns across vignettes. We asked participants to answer them in two conditions: (1) a private one, in which they had to decide privately, and (2) a social one, in which their decisions reflected their own and an additional person fate—for example, a ski trip you accidentally bought was for you and another person, and who offloaded the decision to you.

### 3.1. Participants

We analysed the data of 197 English native speakers, recruited from Prolific Academic with the use of the Qualtrics survey platform, with N = 100 in the private condition and N = 97 in the social condition. Informed consent was obtained from all subjects involved in the study. The sample consisted of 86 males, 109 females, and 2 participants who self-classified as “other”. Their average age was 41.3 (SD = 14.0, range 18–75).

As in Experiment 1, we rejected data from an additional 3 participants, who reported having died of a heart attack while watching Netflix, suggesting their lack of attentiveness to the questions asked.

### 3.2. Materials and Procedure

We presented participants with sunk cost vignettes in a fixed order as shown in Table 2—four from Arkes and Blumer [1], two from Soman [23], and six we designed ourselves to reflect the general pattern of honouring sunk costs. Our vignettes reflected daily, low-stakes decisions which we hoped would better reflect people’s everyday experiences.

The vignettes were presented in their traditional (i.e., private) form to half of the participants. The other half read the vignettes, changed to public versions, oftentimes reflecting other people’s trust in their decisions. To illustrate, the public version of the Michigan ski trip read as follows:

*Assume that you have spent $100 on a ticket for a weekend ski trip to Michigan for you and your partner. You have already informed them about it and they are on the board. Several weeks later, you buy a $50 ticket for a weekend ski trip to Wisconsin. You think you will enjoy the Wisconsin ski trip more than the Michigan ski trip. As you are putting your just-purchased Wisconsin ski trip ticket in your wallet, you notice that the Michigan ski trip and the Wisconsin ski trip are for the same weekend! It’s too late to sell either ticket, and you cannot return either one. You must use one ticket and not the other. Which ski trip will you go on?*

As in Experiment 1, the decisions were not incentivised. The survey was complemented with additional measures: the cognitive reflection test, and self-reports about their financial status, economic knowledge, and knowledge about the issues mentioned in the survey. This time, we did not use the social desirability scale.

### 3.3. Results

Before comparing conditions with a *t*-test, we examined whether the sum scores were justified (Table 3). We performed an analysis for all vignettes, including our own. The results showed reliability estimates not crossing 0.2, far from acceptable values of 0.7–0.8. To ensure that low reliability was not driven by our new items, we recalculated reliability using only previously published vignettes. The results showed that the 6-item long scale constructed from those vignettes would also be unacceptably unreliable. That said, the results showed that the vignettes designed to test one underlying phenomenon—propensity to honour sunk cost—were likely measuring different constructs.

Importantly, poor reliability was observed within each condition separately (public: ω = 0.34/0.14; private: ω = 0.13/0.18), indicating that framing differences between conditions could not account for the weak intercorrelations between vignettes. The measurement problems existed within each framing context, not merely between them.

**Figure 2:**
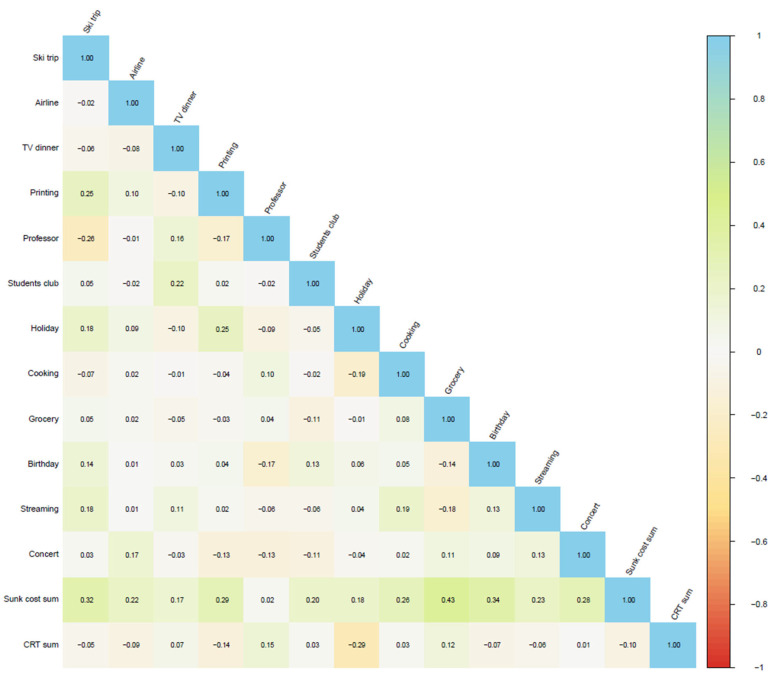
Intercorrelations heatmap for Experiment 2. Note: Correlations are measured by Kendall’s Tau-b.

As in Experiment 1, we investigated the intercorrelations of the items and correlations to the results of the cognitive reflection test (Figure 2). The latter correlations ranged from positive 0.15 to 0.01 to negative 0.29. So, the expected pattern between low cognitive reflection and the propensity to honour sunk costs was unconfirmed here.

Our hypothesis that scenarios would show better intercorrelations was decisively unsupported—the reliability remained poor across all conditions. Similarly, our speculation that social framing could increase correlations through consistent reputation concerns was not confirmed, as reliability was equally poor in both private and public conditions.

Had we ignored the reliability issues, we could infer that participants in the public condition declared a lower willingness to honour sunk costs. However, this conclusion is, in our opinion, unwarranted at the current stage.

## 4. General Discussion

Across the two experiments involving nearly 400 participants, we documented a measurement problem in sunk cost research: vignettes designed to assess the same psychological construct showed poor intercorrelations (ω = 0.14–0.57) and inconsistent relationships with theoretically relevant external measures. This pattern held across different experimental conditions and remained stable when controlling for participant attention and response time, revealing measurement issues that may have impeded theoretical progress for decades.

Our findings suggest three possible interpretations with different implications for sunk cost theory and neuropsychological research. First, people may possess stable individual differences in sunk cost tendencies that current vignettes fail to capture reliably. This interpretation assumes the construct validity of sunk cost bias as a unitary phenomenon but questions current measurement approaches. Under this view, the poor reliability reflects methodological inadequacy rather than theoretical problems, suggesting that better-designed vignettes or alternative assessment methods could reveal consistent individual differences.

Second, different scenarios may tap distinct psychological mechanisms—such as loss aversion, social signalling, or commitment consistency—that have been incorrectly lumped under the umbrella term “sunk cost bias”. This interpretation questions the construct validity of sunk cost bias as traditionally conceived, suggesting that apparent measurement failure actually reflects genuine psychological diversity. The significant correlations that emerged primarily within narrow contextual domains (e.g., airline company scenarios correlating r = 0.32) support this interpretation.

Third, sunk cost behaviour may be primarily context-dependent, with minimal stable individual differences across situations. This interpretation suggests that the search for trait-like measures may be misguided, as sunk cost decisions depend more on situational factors than stable dispositions.

The inconsistent relationship between cognitive reflection and sunk cost scenarios provides evidence for construct heterogeneity. If these vignettes measured a unified psychological construct, we would expect consistent correlations with the CRT across scenarios. Instead, we observed that only the Michigan scenario correlated with the CRT in Experiment 1 (r = −0.17), while only the holiday/lottery scenario showed this relationship in Experiment 2 (r = −0.29). This pattern suggests that cognitive control processes—theoretically central to overcoming sunk cost bias—are relevant to only specific scenario types. Some vignettes may engage deliberative processes that the CRT captures, while others may tap automatic responses, social concerns, or emotional attachments that operate independently of cognitive reflection. This scenario-specific variation in CRT relationships undermines the assumption that all sunk cost vignettes assess the same underlying decision-making process.

While we cannot definitively distinguish between these interpretations, the low correlations between even highly similar vignettes (e.g., different versions of the airline scenario) suggest context plays a larger role than previously recognised. Researchers should investigate a greater number of vignettes and possibly behavioural tasks to classify them into similar groups. Only then can we move forward and establish the underlying psychological and neural mechanisms responsible for the sunk cost bias.

### 4.1. Theoretical Implications: Behavioural Similarity and Psychological Diversity

A crucial insight from our findings is that identical behaviours can stem from different psychological motives. Much like eating can be driven by hunger or boredom—appearing behaviourally identical but requiring different explanations and interventions—people who honour sunk costs may do so for entirely different reasons across contexts. This distinction is critical for scientific understanding because the same observable behaviour (continuing a failing investment) can mask important psychological heterogeneity. When people honour sunk costs, this single behavioural outcome can be driven by multiple distinct psychological processes—each requiring different theoretical explanations and interventions:

- Loss aversion: “I cannot bear to accept this loss”
- Commitment consistency: “I must stick to my decisions to maintain my self-concept”
- Social signalling: “Others will think I am persistent and reliable”
- Optimistic belief updating: “My prior investment increases the likelihood of eventual success”
- Temporal discounting: “I have already invested so much time, a little more won’t matter”

Arkes and Blumer [1] explicitly aimed to demonstrate a general decision-making bias that would manifest consistently across diverse contexts. They wrote that people systematically “throw good money after bad” due to a unified cognitive error, presenting their vignettes as equivalent measures of the same underlying irrationality. Their theoretical framework assumed these scenarios would correlate because they all involve the same systematic violation of economic rationality—ignoring sunk costs when making forward-looking decisions.

However, our reliability analysis challenges this fundamental assumption. The poor correlations between vignettes suggest that people who honour sunk costs in business contexts (airline scenarios) do not necessarily do so in personal contexts (ski trips), indicating these scenarios may tap different psychological motivations rather than a unified bias. A person might be highly susceptible to loss aversion in financial decisions but largely immune to social signalling concerns in recreational choices, leading to apparently inconsistent “sunk cost” behaviour across contexts.

This behavioural–psychological disconnect has profound implications for theory development. If we cannot empirically distinguish between different psychological motives underlying similar behaviours, we cannot build coherent theoretical accounts of when and why sunk cost effects emerge. Current measurement approaches provide no way to determine whether someone who honours sunk costs in one scenario will do so in another, or whether their decisions stem from the same underlying psychological process.

The field needs systematic empirical research to classify scenarios by their underlying psychological mechanisms before meaningful theoretical progress can occur. Our findings suggest that apparent inconsistencies in the sunk cost literature may reflect genuine psychological diversity rather than measurement error, but without reliable tools to map behaviours onto psychological processes, this distinction remains untestable.

In this light, our results suggest that the second interpretation from our discussion is most likely correct: different scenarios do tap distinct psychological mechanisms, and researchers are partially aware of this diversity. However, the field has continued using vignettes interchangeably, perhaps hoping that any underlying differences would average out or prove inconsequential. Our reliability analysis demonstrates that these differences are substantial enough to undermine measurement validity and impede theoretical progress.

Rather than arguing that existing vignettes are “useless”, we suggest they may be quite valuable for studying specific psychological mechanisms—but only if we can empirically map which vignettes tap which processes. The poor intercorrelations we observe may actually be informative, indicating that we need to develop a taxonomy of sunk cost scenarios based on their underlying psychological mechanisms rather than treating them as interchangeable measures of a unified construct.

### 4.2. Neuropsychological Implications

Our findings reveal fundamental measurement problems that have implications for neuroimaging research on sunk cost bias. The poor intercorrelations between vignettes suggest that different scenarios may activate distinct neural networks rather than measuring a unified psychological construct. This measurement heterogeneity undermines the interpretation of existing neuroimaging studies and highlights the need for paradigm shifts in how we study sunk cost behaviour at the neural level.

Valid neuroscientific interpretation requires reliable behavioural measurement, yet our findings demonstrate that widely used sunk cost vignettes lack this prerequisite reliability. When behavioural measures show poor convergent validity, apparent differences in neural activation between studies may reflect the use of vignettes that engage fundamentally different psychological processes rather than genuine theoretical disagreements about sunk cost mechanisms. For example, studies using financial loss scenarios versus time investment vignettes may recruit different neural circuits—not because they measure different aspects of sunk cost bias, but because they tap entirely different psychological processes.

The inconsistent relationship between cognitive reflection and sunk cost scenarios across our experiments exemplifies this problem. This pattern suggests that the documented relationship between prefrontal cognitive control and sunk cost resistance depends heavily on scenario context, potentially explaining contradictory findings in the neuroimaging literature.

Neuroimaging studies have identified key brain regions involved in sunk cost decisions, with evidence pointing to ventromedial prefrontal cortex (vmPFC) and dorsolateral prefrontal cortex (dlPFC) interactions. However, our reliability findings suggest these apparently different neural mechanisms may reflect measurement heterogeneity The poor correlation between experiential scenarios (Michigan ski trip) and business decisions (airline company; r = −0.02) may indicate that these vignettes activate different neural networks: experiential decisions engaging limbic emotion-processing regions versus business decisions recruiting prefrontal control areas. If different vignettes systematically recruit different neural circuits, then treating them as interchangeable measures of “sunk cost bias” conflates multiple distinct neurobiological processes.

The cross-species research conducted by Sweis et al. [11] offers a promising solution to these measurement problems. Their foraging tasks demonstrated reliable sunk cost effects across mice, rats, and humans using identical temporal investment paradigms, with consistent neural activation in the anterior cingulate cortex across species. This suggests that evolutionarily conserved mechanisms can be reliably measured when using appropriate behavioural tasks that capture actual investment and persistence rather than hypothetical scenarios.

Crucially, these cross-species paradigms show robust psychometric properties that current vignette-based measures lack. The behavioural convergence, combined with evidence for shared neural substrates in decision-making circuits, suggests that measurement problems in human vignette-based research reflect methodological limitations rather than the absence of an underlying neurobiological construct. The development of human behavioural tasks that parallel these cross-species paradigms could provide the reliable individual difference measures necessary for meaningful neuroimaging research.

### 4.3. Methodological Recommendations

These findings have implications for neuropsychological research practices. First, researchers conducting neuroimaging studies should report psychometric properties of behavioural measures and justify vignette selection based on demonstrated reliability rather than on face validity. Single-scenario neuroimaging studies should acknowledge limited generalisability and avoid broad claims about the neural mechanisms of “sunk cost bias” based on single-vignette evidence.

Second, the field needs systematic validation studies examining which behavioural paradigms show both adequate psychometric properties and meaningful neural correlates. The contradiction between Negrini et al.’s [3] finding of reverse sunk cost effects with real money versus standard effects with hypothetical scenarios suggests that neural activation patterns may differ substantially between incentivised and hypothetical measures. Monetary incentives may not be the only factor explaining differences between incentivised and hypothetical measures. If sunk cost effects are as contextually sensitive as our reliability analysis suggests, then numerous factors beyond monetary stakes could account for behavioural changes between studies—including social context, task framing, individual differences in scenario relevance, and the specific psychological mechanisms each paradigm engages. This sensitivity to contextual factors further supports our argument that apparent contradictions in the literature may reflect genuine measurement heterogeneity rather than random error.

Third, future research should develop behavioural tasks that translate effectively across species and show robust individual differences. Real effort tasks, temporal investment paradigms, or field studies involving genuine investments may provide more reliable assessment approaches than hypothetical vignettes. Such measures might better capture the underlying psychological mechanisms while reducing the context dependency that appears to plague current approaches.

Finally, computational modelling combined with neuroimaging could help decompose sunk cost behaviour into component processes with distinct neural signatures. Machine learning approaches applied to both behavioural and neural data might identify latent factors that explain the low correlations between vignettes while revealing consistent neural mechanisms across contexts.

### 4.4. Limitations

Several limitations warrant consideration. Our studies used online samples and binary response formats, which may not generalise to other populations or response scales. Our focus on established vignettes means we cannot rule out that newly developed scenarios with stronger theoretical foundations might show better psychometric properties. Additionally, the neural evidence we cited came from studies using different paradigms than the vignettes we tested, limiting direct comparisons between behavioural and neural findings. While our findings raise questions about the reliability of established measures, we acknowledge that our results are limited to the specific vignettes tested and may not generalise to all possible sunk cost assessments.

### 4.5. Conclusions

Our findings reveal measurement challenges in sunk cost research that may have important implications for neuropsychological science. Rather than arguing that existing sunk cost vignettes are fundamentally flawed or useless, our findings suggest they may be valuable tools for studying specific psychological mechanisms—but only if we can empirically classify them by the processes they engage.

The poor intercorrelations we observed may actually be informative, revealing psychological boundaries rather than random measurement error. The field’s intuitions about psychological diversity appear to be correct; our contribution is demonstrating that this diversity has measurable consequences that require systematic investigation.

This suggests a path forward: systematic psychometric validation combined with theoretical analysis to develop a taxonomy of sunk cost scenarios based on their underlying psychological mechanisms. Perhaps most importantly, our findings suggest that the field’s long-standing intuitions about psychological diversity in sunk cost behaviour are not only correct but have measurable consequences that can no longer be ignored. The path forward requires embracing this complexity rather than assuming it away.

## Figures and Tables

**Table 1:** Vignette characteristics and reliability analysis.

Scale Reliability ω = 0.57				If Item Dropped
	Mean	SD	Item–Rest Correlation	McDonald’s ω
Airline company: decide	0.79	0.41	0.43	0.48
Airline company: suggest	0.37	0.49	0.43	0.47
TV dinner	0.75	0.43	−0.05	0.63
Printing press	0.42	0.50	0.01	0.62
Acme: decide	0.69	0.46	0.53	0.43
Acme: suggest	0.39	0.49	0.39	0.48
Michigan	0.31	0.47	−0.05	0.63

**Table 2:** Private versions of vignettes used in Experiment 2.

Label [Source]	Vignette
Ski trip [1]	Assume that you have spent $100 on a ticket for a weekend ski trip to Michigan. Several weeks later you buy a $50 ticket for a weekend ski trip to Wisconsin. You think you will enjoy the Wisconsin ski trip more than the Michigan ski trip. As you are putting your just-purchased Wisconsin ski trip ticket in your wallet, you notice that the Michigan ski trip and the Wisconsin ski trip are for the same weekend! It’s too late to sell either ticket, and you cannot return either one. You must use one ticket and not the other. Which ski trip will you go on? $100 ski trip to Michigan $50 ski trip to Wisconsin
Airline [1]	As the president of an airline company, you have invested 10 million dollars of the company’s money into a research project. The purpose was to build a plane that would not be detected by conventional radar, in other words, a radar-blank plane. When the project is 90% completed, another firm begins marketing a plane that cannot be detected by radar. Also, it is apparent that their plane is much faster and far more economical than the plane your company is building. The question is: should you invest the last 10% of the research funds to finish your radar-blank plane? Yes No
TV dinner [1]	On your way home you buy a tv dinner on sale for $3 at the local grocery store. A few hours later you decide it is time for dinner, so you get ready to put the tv dinner in the oven. Then you get an idea. You call up your friend to ask if he would like to come over for a quick tv dinner and then watch a good movie on tv. Your friend says “Sure.” So you go out to buy a second tv dinner. However, all the on-sale tv dinners are gone. You therefore have to spend $5 (the regular price) for the tv dinner identical to the one you just bought for $3. You go home and put both dinners in the oven. When the two dinners are fully cooked, you get a phone call. Your friend is ill and cannot come. You are not hungry enough to eat both dinners. You can not freeze one. You must eat one and discard the other. Which one do you eat? $5 dinner $3 dinner
Professor [23]	Imagine that you recently saw an advertisement on the bulletin board. A literature professor was looking for a research assistant to work for about 15 h. The payment was in the form of a front row seat to a professional theatre performance. On the same bulletin board, a music professor was also looking for a research assistant to work for about 5 h and this assistant would be paid with a ticket in a good section) to a rock concert by a band that you like. You had recently seen posters for both the theatre performance and the rock concert. You think you will like to see both these events, although you expect to like the rock concert more. You work for both the professors 15 h for literature and 5 h for music) and get paid with the two tickets theatre and rock concert respectively). As you are putting the tickets away in your wallet, you notice that both events are scheduled for the same evening and are both at good locations on campus. The tickets are non-transferable, nor can they be exchanged. You can use only one of the tickets and not the other. Which ticket will you use? The ticket for the theatre performance The ticket for the rock concert
Printing [1]	As the owner of a printing company, you must choose whether to modernize your operation by spending $200,000 on a new printing press or on a fleet of new delivery trucks. You choose to buy the press, which works twice as fast as your old press at about the same cost as the old press. One week after your purchase of the new press, one of your competitors goes bankrupt. To get some cash in a hurry, he offers to sell you his computerized printing press for $10,000. This press works 50% faster than your new press at about one-half the cost. You know you will not be able to sell your new press to raise this money, since it was built specifically for your needs and cannot be modified. However, you do have $10,000 in savings. The question is should you buy the computerized press from your bankrupt competitor? Yes No
Students’ club [23]	You are planning to submit an entry to the `new invention’ competition organized by the students’ club. You have spent 30 h preparing a design for an innovative rocket engine and estimate that it will take you an additional 10 h to finish it. You just learn that the winner of the previous year’s competition was also working on a rocket engine design similar to yours. You had also thought about working on a equally innovative and good) design for a solar powered pump that would take about 10 h to complete. You can submit only one entry, and since the deadline is very close, you must choose now. The question is: Should you spend 10 h trying to finish your rocket engine design? Yes No
Holiday/lottery	You have booked a holidays trip. After having finalized all the payments, you received an information that you have won a trip in a lottery and its destination is much more desirable. However, it’s going to happen in the same time as your intended holidays and if you cancel your first trip, you won’t get a full refund. Would you rather: Go on the trip you have booked Go on the trip you have won in the lottery
Cooking	You have signed up for an eight weeks long cooking course (one meeting a week). During the first few meetings you haven’t learnt anything new, so you asked the teacher about their plans for the future classes; it turns out you already know how to prepare all the dishes you were supposed to learn. Therefore, there is no reason for you to participate in the future meetings, however, you have already paid for them in advance and it’s not possible to get a refund. Would you rather: Drop out of the course Complete the course
Grocery	You are in a grocery store. You have spent the last 10 min waiting in the queue before the cash register. You realize that the queue before the other cash register seems to move much faster than yours. Would you rather: Stay in the queue you’re already standing in Move to the other queue
Birthday	Your friend is throwing a birthday party in a nice restaurant. You know there is going to be champagne, so you decide to get there using public transport instead of driving. However, your bus is significantly late and you start worrying whether it will arrive at all. The restaurant is within the walking distance; if you decided to walk, you would be there on time, but you would have to leave the bus stop immediately. Would you rather: Wait for the bus Walk to the restaurant
Streaming	The streaming service you use has an algorithm choosing series you might like, based on what you have previously watched. Recently, it recommended you a series that already has three seasons, and you know there are more to come. You have already finished one season, and you don’t find it interesting (though you don’t think it’s horrible either). Would you rather: Continue watching this series Stop watching this series
Concert	You have bought tickets for a concert in a city quite far away from where you live. You knew you were going to spend a night there, so you booked a hotel room and decided that, aside from going to the concert, you will also go sightseeing and have a meal in a nice restaurant and although you expect it to be nice, you realize that the concert is the reason you want to travel there. Suddenly, a few days before the date of the concert, you find out it has been cancelled due to the illness of the vocalist. Would you rather: Cancel your trip Go on your trip

Note: Scenarios with no referenced source are self-created by the authors.

**Table 3:** Reliability measures of the private and public versions of the vignettes measuring the propensity to honour sunk cost.

	Public				Private			
Only Old Items (1–6)	ω = 0.34			If Item Dropped	ω = 0.13			If Item Dropped
All Items	ω = 0.14				ω = 0.18			
	Mean	SD	Item–Rest Correlation	McDonald’s ω	Mean	SD	Item–Rest Correlation	McDonald’s ω
Ski trip	0.52	0.50	0.01	0.10	0.29	0.46	0.14	0.11
Airline	0.79	0.41	−0.15	0.22	0.74	0.44	0.09	0.16
TV dinner	0.76	0.43	0.15	0.02	0.21	0.41	0.02	0.18
Professor	0.15	0.36	0.27	−0.05	0.84	0.37	−0.16	0.26
Printing	0.40	0.49	−0.02	0.17	0.43	0.50	0.06	0.15
Students’ club	0.53	0.50	0.09	0.06	0.41	0.49	−0.01	0.19
Holiday/lottery	0.13	0.33	0.02	0.12	0.17	0.38	0.04	0.17
Cooking	0.64	0.48	0.05	0.08	0.67	0.47	0.04	0.18
Grocery	0.43	0.50	−0.12	0.20	0.48	0.50	−0.05	0.22
Birthday	0.05	0.22	−0.10	0.19	0.08	0.27	0.11	0.14
Streaming	0.23	0.42	0.02	0.14	0.20	0.40	0.13	0.12
Concert	0.68	0.47	−0.04	0.18	0.74	0.44	0.02	0.18

## Data Availability

All data and materials are publicly available at the Open Science Framework repository: https://osf.io/ubzv2/?view_only=63adfcdc96924ad68d814e086e816053 (accessed on 27 July 2025).
